# Association of epidural block analgesia combined with Low-dose oxytocin with pain intensity and delivery outcomes in primiparous women

**DOI:** 10.3389/fped.2026.1850211

**Published:** 2026-09-10

**Authors:** Yuhong Zeng, Chaoxiang Shi, Manying Liu, Quanquan Peng

**Affiliations:** Department of Obstetrics and Gynecology, Ganxian District People’s Hospital, Ganzhou City, Jiangxi Province, China

**Keywords:** delivery outcomes, duration of labor, epidural analgesia, labor analgesia, low-dose oxytocin, primiparous women

## Abstract

**Background:**

Epidural block is an effective labor analgesia method; adjunctive low-dose oxytocin may further influence uterine dynamics and clinical outcomes, but evidence in primiparas is limited.

**Objective:**

To evaluate the association of epidural analgesia combined with low-dose oxytocin with pain intensity, labor duration, delivery mode, and immediate maternal–neonatal outcomes in primiparous women.

**Methods:**

In this retrospective study, 120 eligible primiparous women who delivered between January 2023 and January 2025 were classified by analgesia regimen into a combination group (epidural analgesia plus low-dose oxytocin) or a control group (epidural analgesia alone). We compared maternal demographics, VAS pain scores, durations of each labor stage, delivery mode, postpartum blood loss, maternal adverse events, and short-term neonatal outcomes (Apgar scores, NICU admission).

**Results:**

The combination group showed markedly lower VAS scores at 30 min after analgesia, in active labor, and 2 h postpartum (3.1 ± 0.9 vs. 7.0 ± 1.0; 3.5 ± 1.0 vs. 6.8 ± 1.1; 2.8 ± 0.8 vs. 6.5 ± 1.0; all *P* < 0.001). First-stage, second-stage and total labor durations were shorter in the combination group (all *P* < 0.05). Vaginal delivery rate was higher (86.7% vs. 60.0%, *P* = 0.001) and emergency cesarean lower (10.0% vs. 30.0%, *P* = 0.006); combined treatment remained associated with vaginal birth (adjusted OR = 1.92, 95% CI = 1.10–3.34, *P* = 0.02). Common short-term maternal adverse events did not differ significantly between groups(*P* > 0.05), but hospital stay was shorter with the combination (*P* = 0.001).

**Conclusions:**

Epidural analgesia combined with low-dose oxytocin was associated with lower labor pain scores, shorter labor duration, and a higher rate of vaginal delivery in primiparous women, without a detectable increase in common short-term maternal or neonatal adverse events.

## 1 Introduction

Epidural anesthesia, a type of central neuraxial block, entails the administration of local anesthetic agents into the epidural space to inhibit nociceptive signal conduction. This technique is widely acknowledged as the principal modality for labor analgesia (1, 2). Worldwide, it has been established as the reference standard for pain control during childbirth and is consistently recommended by international clinical guidelines due to its ability to enhance maternal comfort while ensuring neonatal safety during spontaneous labor (3, 4). Recently, innovative approaches, including low-dose protocols, patient-controlled epidural analgesia (PCEA), and programmed intermittent epidural bolus techniques, have been introduced to improve analgesic effectiveness and maternal satisfaction, concurrently decreasing total anesthetic exposure and minimizing the likelihood of motor impairment (5, 6). Low-dose oxytocin therapy is commonly used during labor to promote uterine contractions and accelerate labor progression, typically administered via continuous low-dose intravenous infusion. Its safety and clinical advantages have been increasingly recognized (7, 8). Evidence suggests that low-dose oxytocin protocols achieve comparable effects to high-dose regimens in shortening labor duration and reducing postpartum hemorrhage, while minimizing adverse events such as uterine hyperstimulation (7, 8). Primiparas—women experiencing labor for the first time with a term, singleton pregnancy—often exhibit slower cervical dilation, prolonged labor, and higher pain perception, making them a key target group for analgesia and labor management (9, 10). In the delivery or labor ward, comprehensive labor management emphasizes strict adherence to standardized procedures and monitoring protocols, encompassing analgesic techniques, pharmacologic management, and continuous assessment of maternal and neonatal vital signs (3, 11). Maternal labor pain triggers stress responses that may hinder the normal progression of labor and amplify emotional strain. Evidence from multiple investigations indicates a significant association between the intensity of pain and levels of psychological distress, highlighting the critical multidimensional role of adequate pain management during childbirth (12, 13). Epidural analgesia effectively alleviates pain intensity, enhances maternal comfort, and reduces stress-related biomarkers, without significant short-term adverse effects on maternal or neonatal outcomes (1, 6). As a uterotonic agent, oxytocin can regulate the amplitude and frequency of uterine contractions during continuous infusion. When appropriately dosed, it facilitates labor progression without significantly increasing the rate of cesarean section or adverse maternal-fetal events (8, 14). Based on this evidence, the present retrospective cohort study focuses on primiparous women to systematically evaluate the association between epidural analgesia combined with low-dose oxytocin and labor pain, labor duration, delivery mode, intervention rates, and maternal-neonatal outcomes. We hypothesize that the combined approach may be associated with lower pain scores and improved labor-related outcomes without increasing the risk of adverse events. Through population stratification and multidimensional outcome analysis, this study aims to provide evidence-based support and innovative insights for clinical decision-making in labor analgesia and oxytocin administration strategies.

## 2 Methods

### 2.1 Study design and participants

This investigation was conducted as a single-center retrospective cohort analysis, enrolling primiparous women who delivered at our hospital. from January 2023 to January 2025. All participants received epidural analgesia and were categorized into two groups according to documentation of adjunctive low-dose oxytocin administration in the medical records: the combination group (epidural analgesia with low-dose oxytocin) and the control group (epidural analgesia alone). The study was conducted in accordance with the STROBE (Strengthening the Reporting of Observational Studies in Epidemiology) statement. Because this study was based on de-identified data extracted from existing medical records and involved no additional intervention, participant contact, or collection of biological specimens, the hospital ethics committee granted an exemption from formal ethical review and waived the requirement for written informed consent.

### 2.2 Inclusion and exclusion criteria

The inclusion criteria were as follows: ① Age between 18 and 45 years, singleton pregnancy, cephalic presentation, and primiparous status; ② Gestational age ≥37 weeks, with indications for vaginal delivery and receipt of epidural analgesia at our hospital; ③ Complete medical records with available data on anesthesia, labor, and neonatal outcomes. Exclusion criteria included: ① History of cesarean section or pelvic surgery; ② Multiple pregnancies or non-cephalic presentation; ③ Significant impairment of cardiac, liver, or kidney function, or any other condition influencing the metabolism of medications; ④ Use of medications during labor inconsistent with the study interventions; ⑤ Incomplete or missing medical records preventing retrieval of primary study outcomes.

### 2.3 Interventions

All participants received a standardized regimen of epidural analgesia. An epidural catheter was placed at either the L2–3 or L3–4 interspace, followed by a test dose of 3 mL of 1.5% lidocaine. Subsequently, a loading dose consisting of 0.1%–0.125% ropivacaine or bupivacaine in combination with a low-concentration opioid was administered. Analgesia was maintained using patient-controlled epidural analgesia (PCEA) with a continuous basal infusion of 6–10 mL/h, a patient-initiated bolus of 5–8 mL, and a lockout interval ranging from 10 to 20 min.

According to the medical records, in all women included in the combination group, oxytocin was initiated after epidural analgesia had been established. The infusion was initiated at 1–2 mU/min and titrated progressively according to uterine contraction patterns and fetal heart rate monitoring, with a maximum allowable rate of 6 mU/min. Administration followed institutional guidelines, which indicated oxytocin for scenarios such as cervical dilation less than 1 cm per hour, labor stagnation, or uterine hypotonia. The indication for oxytocin administration was determined by the attending obstetrician based on real-time assessment of labor progress and uterine contraction adequacy. In general, oxytocin was started after epidural analgesia had been established and maternal hemodynamics and fetal heart rate patterns were confirmed to be stable. The duration of oxytocin infusion was individualized according to labor progression, uterine contraction patterns, and fetal monitoring findings, and infusion was discontinued or adjusted when adequate contractions were achieved or when uterine hyperstimulation or abnormal fetal heart rate patterns occurred. Aside from these interventions, all other aspects of labor management adhered to standard institutional protocols.

### 2.4 Observation indicators

In this study, maternal pain intensity and delivery outcomes were designated as co-primary endpoints. Maternal pain was evaluated using the Visual Analogue Scale (VAS; 0 = no pain, 10 = maximum pain). VAS scores were recorded at four time points: before analgesia, 30 min after analgesia, during the active phase of labor (cervical dilation ≥ 6 cm), and 2 h postpartum. A VAS score > 6 after analgesia was regarded as clinically relevant residual pain rather than a satisfactory analgesic target. Bishop score at admission referred to the assessment performed at admission to the labor ward before epidural analgesia initiation. Delivery outcome was defined by the final mode of delivery, categorized as vaginal delivery (spontaneous or assisted) or cesarean section (elective or emergency). The primary indications for cesarean section—such as fetal distress and labor arrest—were also documented. Secondary endpoints included: ① Duration of labor: The first stage was defined as the interval from the documented onset of regular uterine contractions after admission to the labor ward to full cervical dilation, the second stage as the interval from full cervical dilation to fetal expulsion, and the total labor duration as the interval from the documented onset of regular uterine contractions after admission to the labor ward to delivery. All labor durations were recorded in minutes. ② Maternal safety and perinatal complications: Postpartum blood loss was quantified using a combination of weighing and blood collection methods, expressed in milliliters (mL). Incidences of postpartum hemorrhage (>1,000 mL) and blood transfusion were recorded. Other complications included transient hypotension (systolic blood pressure decrease > 20% or <100 mmHg), nausea and vomiting, uterine hyperstimulation, and epidural-related complications (headache, infection, hematoma), as well as any other adverse events requiring medical intervention. ③ Neonatal short-term outcomes: Neonatal status was evaluated by Apgar scores at 1 and 5 min, the need for resuscitation or NICU admission, and incidences of asphyxia or other severe complications. ④ Length of maternal and neonatal hospital stay (in days) was also evaluated.

### 2.5 Data sources and collection

All information utilized in this retrospective study was retrieved from the hospital's electronic medical record (EMR) system, encompassing anesthesia documentation, labor and delivery logs, and neonatal records. Data extraction was independently conducted by two investigators and subsequently cross-checked; any inconsistencies were resolved by a third reviewer. Primary variables of interest included VAS pain scores, labor duration, delivery method, estimated maternal blood loss, and neonatal Apgar scores. Records with missing data for the primary study outcomes or with more than 20% of the required variables unavailable were excluded from the analysis. The completeness and internal consistency of the extracted data were verified before statistical analysis. All patient information was de-identified and securely stored on hospital servers with limited access to maintain confidentiality.

### 2.6 Statistical analysis

Statistical analyses were carried out with IBM SPSS Statistics 26.0 (IBM Corp., Armonk, NY, USA) and R version 4.0 (R Foundation for Statistical Computing, Vienna, Austria). Normally distributed continuous variables were compared between groups using independent-samples t tests and are presented as the mean ± standard deviation. Non-normally distributed continuous variables were compared using the Mann-Whitney U test and are presented as the median and interquartile range. Categorical variables were compared between groups using the chi-square test or Fisher's exact test and are presented as frequencies and percentages. Multivariable binary logistic regression was used to evaluate the association between the combined regimen and vaginal delivery after adjustment for potential confounders, including maternal age, gestational age, Bishop score at labor-ward admission, BMI, pregnancy complications, and estimated fetal weight. Given the limited sample size and the number of non-vaginal delivery events, the primary model was restricted to prespecified clinically relevant covariates, whereas selected intrapartum variables were evaluated in unadjusted between-group analyses to preserve model stability. The results are reported as adjusted odds ratios (ORs) with 95% confidence intervals (CIs). All tests were two-sided, and *P* < 0.05 was considered statistically significant.

## 3 Results

### 3.1 Comparison of clinical baseline and intrapartum characteristics between the two groups

**Figure 1:**
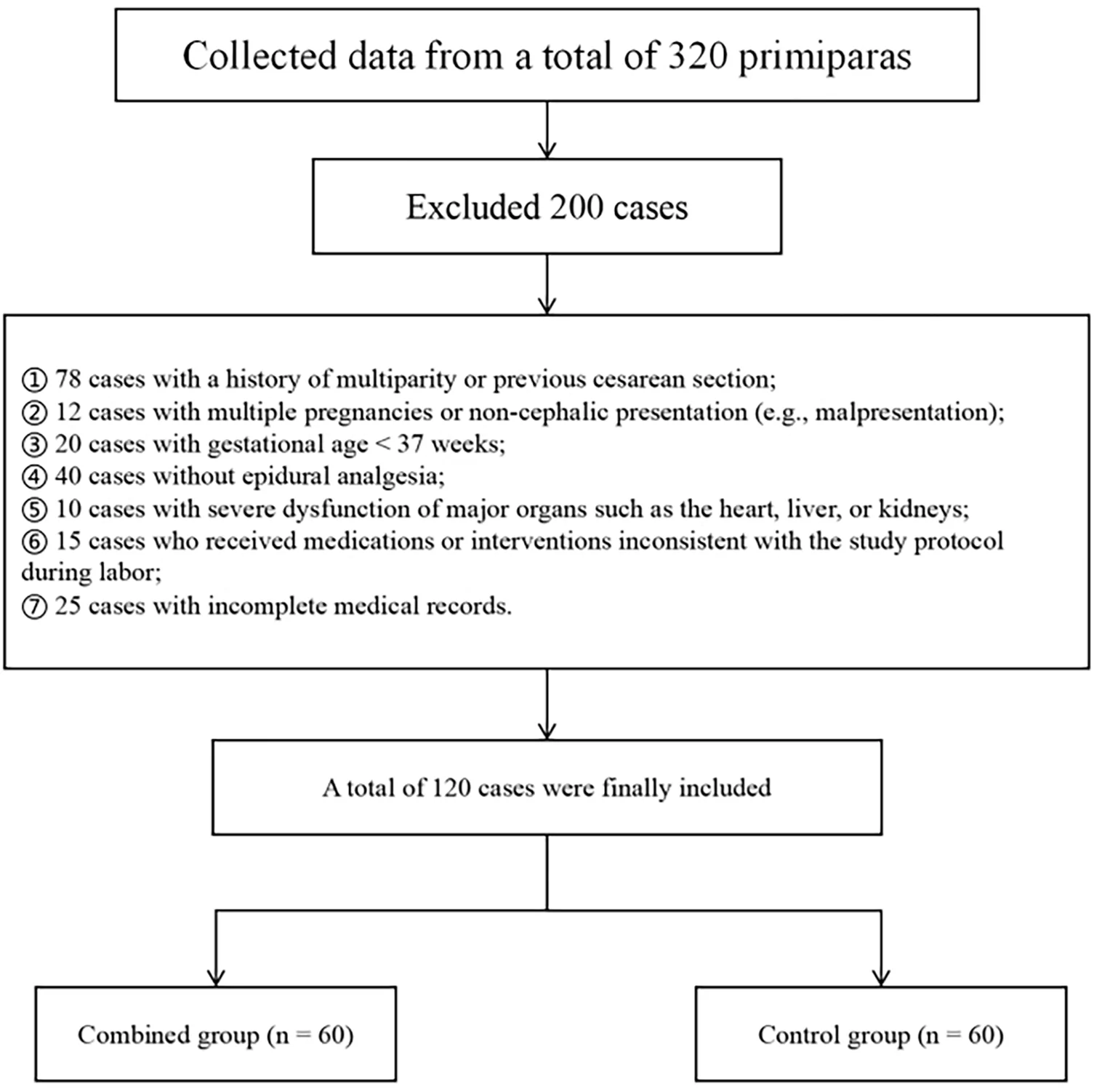
Flowchart of study participant enrollment.

A total of 320 parturition records were initially reviewed for inclusion in this retrospective study. Following stepwise application of the predetermined eligibility criteria, 200 cases were excluded, resulting in a final cohort of 120 primiparous participants. The detailed enrollment and exclusion process is summarized in Figure 1. Based on whether a low dose of oxytocin was administered in combination with epidural block, participants were assigned to either the combination group or the control group, with 60 cases in each group.

Following enrollment, the two groups exhibited no significant differences in key baseline and selected intrapartum characteristics (Table 1). Maternal age (29.1 ± 3.8 vs. 29.8 ± 4.1 years, *P* = 0.334), gestational age at delivery (39.1 ± 0.8 vs. 39.0 ± 0.9 weeks, *P* = 0.521), and body mass index (24.6 ± 3.2 vs. 25.0 ± 3.5 kg/m², *P* = 0.515) were similar across groups. The median Bishop score upon admission was 3 in both cohorts [combination group: 3 [IQR = 2–4] vs. control group: 3 [IQR = 2–5], *P* = 0.529], and estimated fetal weight did not differ significantly (3,340 ± 320 g vs. 3,360 ± 310 g, *P* = 0.729). Moreover, the prevalence of gestational hypertension, gestational diabetes, history of major organ disease, smoking, and other pregnancy-associated complications showed no statistically significant variation between the groups (all *P* > 0.05).

**Table 1:** Comparison of clinical baseline and intrapartum characteristics between the two groups of parturients.

Variable (unit/description)	Combined group (*n* = 60)	Control group (*n* = 60)	Test statistic	*P* value
Age (years), mean ± SD	29.1 ± 3.8	29.8 ± 4.1	*t* = −0.970	0.334
Gestational age (weeks), mean ± SD	39.1 ± 0.8	39.0 ± 0.9	*t* = 0.643	0.521
Body mass index (BMI, kg/m²), mean ± SD	24.6 ± 3.2	25.0 ± 3.5	*t* = −0.653	0.515
Bishop score at labor-ward admission, median (IQR)	3 (2–4)	3 (2–5)	*U* = 1,680	0.529
Estimated fetal weight (g), mean ± SD	3,340 ± 320	3,360 ± 310	*t* = −0.348	0.729
Pregnancy-induced hypertension (PIH), *n* (%)	4 (6.7%)	5 (8.3%)	——	1.000
Gestational diabetes mellitus (GDM), *n* (%)	6 (10.0%)	7 (11.7%)	*χ*^2^ = 0.086	0.769
Pre-existing major organ diseases (cardiac/hepatic/renal), *n* (%)	2 (3.3%)	1 (1.7%)	——	1.000
Smoking history, *n* (%)	0 (0%)	1 (1.7%)	——	1.000
Other major pregnancy complications (total), *n* (%)	7 (11.7%)	8 (13.3%)	*χ*^2^ = 0.076	0.783
Cervical dilation at epidural placement (cm), mean ± SD	3.6 ± 0.9	3.7 ± 1.0	*t* = −0.576	0.566
Time from admission to epidural initiation (min), mean ± SD	158 ± 64	165 ± 70	*t* = −0.572	0.568
Time from regular contractions to epidural initiation (min), mean ± SD	265 ± 95	278 ± 105	*t* = −0.711	0.479
Fetal position at epidural placement, *n* (%)				
- Occiput anterior	45 (75.0%)	43 (71.7%)	*χ*^2^ = 0.18	0.913
- Occiput transverse	10 (16.7%)	11 (18.3%)		
- Occiput posterior	5 (8.3%)	6 (10.0%)		
Fetal station at epidural placement, *n* (%)				
- −3 station	8 (13.3%)	9 (15.0%)	*χ*^2^ = 0.12	0.989
- −2 station	26 (43.3%)	25 (41.7%)		
- −1 station	20 (33.3%)	19 (31.7%)		
- 0 station	6 (10.0%)	7 (11.7%)		

The intrapartum variables were recorded at or before epidural analgesia initiation. Fetal position was categorized according to occiput orientation. Fetal station was assessed by routine obstetric examination. “—” indicates not applicable or not observed.

Selected intrapartum variables recorded at or before epidural analgesia initiation were also well balanced between groups. Cervical dilation at epidural placement was comparable between the combination and control groups (3.6 ± 0.9 vs. 3.7 ± 1.0 cm, *P* = 0.566). Similarly, there were no significant differences in the time from admission to epidural initiation (158 ± 64 vs. 165 ± 70 min, *P* = 0.568) or the time from regular contractions to epidural initiation (265 ± 95 vs. 278 ± 105 min, *P* = 0.479). Fetal position at epidural placement was comparable between groups, with occiput anterior position observed in 75.0% of women in the combination group and 71.7% in the control group (*P* = 0.913). Fetal station at epidural placement was likewise similar between groups (*P* = 0.989).

Overall, these findings suggest that the two groups were comparable with respect to measured baseline and selected intrapartum characteristics, providing a basis for subsequent comparisons of pain-related and delivery outcomes.

### 3.2 Comparison of pain intensity and labor duration between the two groups

Prior to intervention, baseline pain intensity was similar between the groups, with pre-analgesia VAS scores showing no significant difference (7.5 ± 1.1 vs. 7.6 ± 1.0, *P* = 0.603). At 30 min after analgesia, the mean VAS score was significantly lower in the combination group than in the control group (3.1 ± 0.9 vs. 7.0 ± 1.0; mean difference, −3.9 points; *t* = −22.454, *P* < 0.001). Notably, the mean VAS score of >6 in the control group suggested clinically relevant residual pain at this time point rather than adequate analgesic control. During the active phase of labor (cervical dilation ≥ 6 cm) and 2 h postpartum, the combination group demonstrated consistently lower pain scores, recorded as 3.5 ± 1.0 vs. 6.8 ± 1.1 (difference −3.3; *t* = −17.195, *P* < 0.001) and 2.8 ± 0.8 vs. 6.5 ± 1.0 (difference −3.7; *t* = −22.380, *P* < 0.001), respectively. These findings suggest an association between the combined regimen and lower recorded pain scores; however, the retrospective design precludes confirmation of a direct analgesic effect of oxytocin. Regarding labor duration, the combination group also showed shorter times in both the first and second stages. The mean duration of the first stage was 330 ± 100 min, compared with 370 ± 105 min in the control group (difference −40 min; *t* = −2.137, *P* = 0.035). The second stage lasted 80 ± 30 min vs. 98 ± 35 min (difference −18 min; *t* = −3.025, *P* = 0.003), and the total labor duration was significantly reduced in the combination group (420 ± 110 min vs. 480 ± 125 min; difference −60 min; *t* = −2.791, *P* = 0.006) (Table 2).

**Table 2:** Comparison of maternal pain scores and labor durations between the two groups.

Indicator (measurement time/unit)	Combined group (*n* = 60)	Control group (*n* = 60)	*t* value	*P* value
Maternal pain score (VAS, 0–10)				
VAS before analgesia (mean ± SD)	7.5 ± 1.1	7.6 ± 1.0	−0.521	0.603
VAS at 30 min after analgesia (mean ± SD)	3.1 ± 0.9	7.0 ± 1.0	−22.454	<0.001
VAS during active labor (≥6 cm cervical dilation, mean ± SD)	3.5 ± 1.0	6.8 ± 1.1	−17.195	<0.001
VAS at 2 h after delivery (mean ± SD)	2.8 ± 0.8	6.5 ± 1.0	−22.380	<0.001
Labor duration (min)				
First stage of labor (mean ± SD)	330 ± 100	370 ± 105	−2.137	0.035
Second stage of labor (mean ± SD)	80 ± 30	98 ± 35	−3.025	0.003
Total labor duration (mean ± SD)	420 ± 110	480 ± 125	−2.791	0.006

### 3.3 Comparison of delivery outcomes between the two groups

In unadjusted comparisons, the vaginal delivery rate was significantly higher in the combined intervention group than in the control group (52/60, 86.7% vs. 36/60, 60.0%; *χ*^2^ = 10.91, *P* = 0.001), whereas the cesarean section rate was markedly lower in the combined group (8/60, 13.3% vs. 24/60, 40.0%; *χ*^2^ = 10.91, *P* = 0.001). The percentage of natural births in the combined group was significantly higher (44/60, 73.3% vs. 30/60, 50.0%; *χ*² = 6.91, *P* = 0.009), according to additional sub-item analysis, but the rate of instrument-assisted vaginal deliveries was not statistically different between the two groups (13.3% vs. 10.0%, *P* = 0.570). Regarding cesarean section types, the incidence of emergency cesarean sections was higher in the control group (30.0% vs. 10.0%; *χ*^2^ = 7.50, *P* = 0.006), whereas elective cesarean rates were comparable between groups (3.3% vs. 10.0%, *P* = 0.272). Fetal distress (6.7% vs. 16.7%, *P* = 0.153) and labor arrest/poor advancement (5.0% vs. 15.0%, *P* = 0.125) were more common in the control group, according to an itemized comparison of cesarean indications, however neither reached conventional statistical significance. There were few instances of induction failure and other uncommon signs, and there were no discernible intergroup variations (Table 3). Overall, our results suggest that a reduced incidence of emergency cesarean sections and a greater percentage of vaginal deliveries are linked to the combined use of low-dose oxytocin and epidural analgesia.

**Table 3:** Comparison of delivery methods and outcomes between the two groups.

Item	Combined group (*n* = 60)	Control group (*n* = 60)	Test statistic	*P* value
Mode of delivery				
Vaginal delivery — total, *n* (%)	52 (86.7%)	36 (60.0%)	*χ*^2^ = 10.91	0.001
- Spontaneous vaginal delivery, *n* (%)	44 (73.3%)	30 (50.0%)	*χ*^2^ = 6.91	0.009
- Instrument-assisted vaginal delivery, *n* (%)	8 (13.3%)	6 (10.0%)	*χ*^2^ = 0.32	0.570
Cesarean section — total, *n* (%)	8 (13.3%)	24 (40.0%)	*χ*^2^ = 10.91	0.001
- Elective cesarean section, *n* (%)	2 (3.3%)	6 (10.0%)	——	0.272
- Emergency cesarean section, *n* (%)	6 (10.0%)	18 (30.0%)	*χ*^2^ = 7.50	0.006
Indications for cesarean section				
Fetal distress, *n* (%)	4 (6.7%)	10 (16.7%)	——	0.153
Labor arrest/poor progress, *n* (%)	3 (5.0%)	9 (15.0%)	——	0.125
Failed induction, *n* (%)	1 (1.7%)	3 (5.0%)	——	0.620
Maternal request or other non-urgent indications, *n* (%)	0 (0%)	1 (1.7%)	——	1.000
Others (e.g., malpresentation, placenta previa, or complications), *n* (%)	0 (0%)	1 (1.7%)	——	1.000

Instrument-assisted vaginal delivery included vacuum extraction and forceps delivery. “—” indicates not applicable or not observed.

A multivariable binary logistic regression analysis was used to take potential confounding variables into consideration. The combined intervention remained associated with vaginal delivery after controlling for maternal age, gestational age, Bishop score at labor-ward admission, BMI, pregnancy complications, and estimated fetal weight (adjusted OR = 1.92, 95% CI = 1.10–3.34, Wald *z* = 2.30, *P* = 0.02), suggesting that the combined regimen was associated with higher odds of vaginal delivery. The model also showed that a higher Bishop score at labor-ward admission was associated with higher odds of vaginal delivery (per 1-point increase, OR = 1.28, *P* < 0.001), whereas each 100-g increase in estimated fetal weight was modestly associated with lower odds of vaginal delivery (OR = 0.91, *P* = 0.025). Other covariates—including maternal age, gestational age, BMI, and pregnancy complications—did not reach statistical significance in this model (Table 4).

**Table 4:** Multivariable logistic regression for vaginal delivery (adjusted).

Variable (coding/unit)	Regression coefficient (*β*)	OR	95% CI	Wald *z*	*P* value	VIF
Combined medication use (Yes vs. No)	0.65	1.92	1.10–3.34	*z* = 2.30	0.02	1.8
Age (per 1-year increase)	−0.01	0.99	0.93–1.05	*z* = −0.33	0.74	1.2
Gestational age (per 1-week increase)	0.03	1.03	0.86–1.25	*z* = 0.31	0.76	1.3
Bishop score at admission (per 1-point increase)	0.25	1.28	1.12–1.46	*z* = 3.65	<0.001	1.6
BMI (per 1 kg/m² increase)	−0.04	0.96	0.89–1.04	*z* = −1.03	0.30	1.4
Pregnancy complications (Yes vs. No)	−0.30	0.74	0.32–1.70	*z* = −0.71	0.48	1.2
Estimated fetal weight (per 100 g increase)	−0.09	0.91	0.84–0.99	*z* = −2.25	0.025	1.5

### 3.4 Comparison of maternal safety and perinatal complications between the two groups

Regarding substantial bleeding or early postoperative complications, there were no statistically significant differences seen between the two groups. The combined group's mean postpartum blood loss was 358 ± 130 mL, while the control group's was 345 ± 120 mL (*t* = 0.569, *P* = 0.570). Both blood transfusion and postpartum hemorrhage (>1,000 mL) were extremely rare and did not significantly differ across the groups (1.7% vs. 3.3%, *P* = 1.00). The rates of transient hypotension and nausea/vomiting were also comparable (5.0% vs. 6.7%, 6.7% vs. 8.3%, both *P* = 1.00). Uterine hyperstimulation and severe epidural-related complications (such as puncture-site infection or epidural hematoma) were rare or absent, and the incidence of post-dural puncture headache was 1.7% in both groups (*P* = 1.00). Other transient events requiring medical intervention occurred in one case each and resolved after treatment. When these adverse events were combined into a composite endpoint, the incidence was 8.3% in the combined group and 10.0% in the control group, with no significant difference (*χ*^2^ = 0.100, *P* = 0.751). Notably, the length of hospital stay was significantly shorter in the combined group (3.2 ± 0.9 days vs. 3.8 ± 1.0 days, t = −3.45, *P* = 0.001), which may be related to the higher vaginal delivery rate and lower emergency cesarean rate observed in this group (Table 5). Overall, within this sample, the combined use of epidural analgesia and low-dose oxytocin did not increase short-term maternal adverse events.

**Table 5:** Comparison of maternal safety and perinatal complications between Two groups.

Indicator (measurement/definition)	Combined group (*n* = 60)	Control group (*n* = 60)	Test statistic	*P* value
Postpartum blood loss (mL, mean ± SD)	358 ± 130	345 ± 120	*t* = 0.569	0.570
Postpartum hemorrhage (>1,000 mL), *n* (%)	1 (1.7%)	2 (3.3%)	——	1.000
Transfusion rate, *n* (%)	1 (1.7%)	2 (3.3%)	——	1.000
Transient hypotension, *n* (%)	3 (5.0%)	4 (6.7%)	——	1.000
Nausea/vomiting, *n* (%)	4 (6.7%)	5 (8.3%)	——	1.000
Uterine hyperstimulation, *n* (%)	1 (1.7%)	0 (0%)	——	1.000
Epidural-related complications				
Post-dural puncture headache (PDPH), *n* (%)	1 (1.7%)	1 (1.7%)	——	1.000
Epidural puncture site infection, *n* (%)	0 (0%)	0 (0%)	——	——
Epidural hematoma, *n* (%)	0 (0%)	0 (0%)	——	——
Other adverse events requiring medical intervention (description), *n* (%)	1 (1.7%) (transient altered consciousness, recovered after observation)	1 (1.7%) (transient tachycardia, recovered after monitoring)	——	1.000
Composite adverse events (any of the above maternal adverse events), *n* (%)	5 (8.3%)	6 (10.0%)	*χ*^2^ = 0.100	0.751
Length of hospital stay (days, mean ± SD)	3.2 ± 0.9	3.8 ± 1.0	*t* = −3.45	0.001

“—” indicates not applicable or not observed.

### 3.5 Comparison of short-term neonatal outcomes

The short-term prognosis of neonates was generally comparable between the two groups. The mean Apgar scores at 1 and 5 min after birth showed no significant difference between the combined group and the control group (1 min: 8.0 ± 0.7 vs. 7.9 ± 0.8, *t* = 0.729, *P* = 0.468; 5 min: 9.6 ± 0.5 vs. 9.5 ± 0.4, *t* = 1.21, *P* = 0.229). Active resuscitation was required in 1 neonate (1.7%) in the combination group and 2 neonates (3.3%) in the control group (*P* = 1.00). NICU admission occurred in 2 neonates (3.3%) and 3 neonates (5.0%), respectively (*P* = 1.00). No cases of low Apgar scores (≤3), intracranial hemorrhage, or other severe neonatal complications were observed in this study. Birth weight and sex distribution were also comparable between the groups (birth weight: 3375 ± 315 g vs. 3410 ± 330 g, *t* = −0.594, *P* = 0.729; male infants: 51.7% vs. 48.3%, *χ*^2^ = 0.133, *P* = 0.715). The incidence of short-term complications prior to discharge, such as the need for phototherapy or feeding difficulties, was low and showed no significant difference between groups (5.0% vs. 6.7%, *P* = 1.00). Similarly, the length of neonatal hospitalization did not differ significantly between the two groups (3.6 ± 1.2 vs. 3.9 ± 1.5 days, *t* = −1.21, *P* = 0.228) (Table 6). This finding may reflect that neonatal hospitalization was mainly determined by neonatal clinical status, routine postnatal observation, feeding adaptation, and jaundice monitoring, rather than by maternal delivery mode alone. The administration of low-dose oxytocin combined with epidural anesthesia did not appear to exert any detectable adverse effects on short-term neonatal outcomes.

**Table 6:** Neonatal short-term outcomes.

Parameter (Unit/Definition)	Combined group (*n* = 60)	Control group (*n* = 60)	Test statistic	*P* value
1 min Apgar score (mean ± SD)	8.0 ± 0.7	7.9 ± 0.8	*t* = 0.729	0.468
5 min Apgar score (mean ± SD)	9.6 ± 0.5	9.5 ± 0.4	*t* = 1.21	0.229
Need for resuscitation (e.g., intubation, chest compressions), *n* (%)	1 (1.7%)	2 (3.3%)	——	1.000
NICU admission, *n* (%)	2 (3.3%)	3 (5.0%)	——	1.000
Neonatal asphyxia or severe complications (including low Apgar ≤3, intracranial hemorrhage, etc.), *n* (%)	0 (0%)	0 (0%)	——	——
Birth weight (g, mean ± SD)	3375 ± 315	3410 ± 330	*t* = −0.594	0.553
Sex (male), *n* (%)	31 (51.7%)	29 (48.3%)	*χ*^2^ = 0.133	0.715
Short-term complications before discharge (including jaundice requiring phototherapy, feeding difficulties, etc.), *n* (%)	3 (5.0%)	4 (6.7%)	——	1.000
Length of hospital stay (days, mean ± SD)	3.6 ± 1.2	3.9 ± 1.5	*t* = −1.21	0.228

“—” indicates not applicable or not observed.

## 4 Discussion

Epidural analgesia is widely used for labor pain management because of its reliable analgesic efficacy and acceptable maternal-neonatal safety profile. In the present retrospective cohort, epidural analgesia combined with low-dose oxytocin was associated with lower recorded pain scores, shorter labor duration, and a higher vaginal delivery rate in primiparous women. Maternal safety and short-term neonatal outcomes were generally comparable between groups, with no detectable increase in common short-term adverse events. These findings suggest that the combined regimen may be clinically feasible in selected primiparous women, although the observational design precludes causal inference.

Labor pain not only affects maternal psychological well-being but may also interfere with labor progression and maternal-neonatal outcomes, underscoring the clinical importance of effective analgesia (15). Epidural analgesia, due to its reliable analgesic efficacy and high safety profile, has become a standard approach for labor analgesia (16, 17). By blocking nociceptive transmission from the lower body, it can effectively relieve pain in primiparous women and enhance the childbirth experience without increasing the cesarean section rate. In this study, pre-analgesia VAS scores were comparable between the two groups, and all participants received a similar epidural analgesia regimen. However, the control group had a mean VAS score > 6 at 30 min after analgesia, indicating clinically relevant residual pain. This residual pain may be related to variations in intrapartum conditions, uterine contraction intensity, individual response to epidural analgesia, timing of analgesia initiation, or rescue analgesic management. Although cervical dilation at epidural placement and timing of epidural initiation were comparable between groups, the retrospective design limited detailed evaluation of dynamic analgesic adequacy and subsequent rescue interventions. Moreover, oxytocin is primarily a uterotonic agent and may intensify uterine contractions. Therefore, the lower recorded VAS scores in the combination group should be interpreted cautiously and should not be taken as evidence that exogenous oxytocin directly enhanced analgesia. Previous studies have suggested that epidural analgesia combined with oxytocin may be associated with improved labor progression and acceptable short-term maternal and neonatal outcomes, possibly through the uterotonic effect of oxytocin in enhancing uterine contraction efficiency (18). However, because exogenous oxytocin primarily acts as a uterotonic agent and may increase contraction intensity, the present retrospective study cannot confirm a direct analgesic effect of oxytocin or a synergistic analgesic interaction with epidural analgesia.

Delivery outcomes are clinically important indicators in labor management. In the present study, the combination group had shorter first-stage, second-stage, and total labor durations, as well as a higher vaginal delivery rate and a lower emergency cesarean section rate. Prior research indicates that contemporary epidural analgesia methods do not elevate cesarean delivery rates, and the clinical consequences of extended labor appear to be minimal (6). Prolonged labor may be associated with maternal fatigue, fetal distress, and an increased risk of infection; therefore, balancing analgesia with labor progression remains clinically relevant (19). The association observed in this study between the combined regimen and improved delivery outcomes may be partly related to the uterotonic effect of oxytocin and individualized labor management. In addition, optimized epidural analgesia may no longer significantly prolong labor, and its effects on uterine contraction dynamics can be balanced through individualized oxytocin management (6, 20). Oxytocin may improve uterine contraction effectiveness, promote cervical maturation, and accelerate labor progression, thereby contributing to improved delivery outcomes (18). The combined use of epidural analgesia and low-dose oxytocin was associated with improved contraction efficiency and reduced maternal discomfort in this cohort; however, the retrospective design does not allow separation of the independent oxytocin effect from a possible interaction with epidural analgesia.

The safety of interventions plays a central role in assessing maternal and neonatal short-term outcomes and in guiding delivery management. Key clinical endpoints include postpartum hemorrhage, perinatal complications, and neonatal prognosis (21). In the present study, no significant differences were observed between the two groups in postpartum blood loss, postpartum hemorrhage, transfusion, transient hypotension, nausea/vomiting, uterine hyperstimulation, epidural-related complications, neonatal Apgar scores, NICU admission, or short-term neonatal complications. These findings are generally consistent with previous reports indicating that epidural analgesia and appropriately managed oxytocin administration are not clearly associated with increased short-term maternal or neonatal adverse events (22–25). The shorter maternal hospital stay observed in the combination group may be partly related to the higher vaginal delivery rate and lower emergency cesarean section rate in this group. In contrast, neonatal hospital stay did not differ significantly between groups, probably because neonatal discharge was influenced more by neonatal adaptation, feeding status, jaundice monitoring, and institutional observation practices than by maternal length of stay alone. However, the absence of statistically significant differences in adverse events should be interpreted cautiously, because the sample size was limited and most safety outcomes were uncommon. Therefore, the present findings suggest no detectable increase in common short-term maternal or neonatal adverse events in this cohort, but larger prospective studies are needed to evaluate rare or delayed complications.

### Limitations and future perspectives

The primary limitation of this study lies in its retrospective, non-randomized design, which introduces selection bias and residual confounding. Although several known covariates were adjusted for in multivariable analyses, unmeasured or inadequately controlled confounders cannot be excluded. In particular, oxytocin was administered according to clinical indications, such as labor stagnation, inadequate cervical dilation, or uterine hypotonia, rather than by random allocation; therefore, confounding by indication may have influenced both labor duration and delivery mode. Therefore, this study can only indicate an association between epidural analgesia combined with low-dose oxytocin and delivery outcomes, but cannot establish causality or confirm a true synergistic effect between oxytocin and epidural analgesia. Although selected intrapartum variables, including cervical dilation at epidural placement, timing of epidural initiation, fetal position, and fetal station, were compared between groups, they were not all incorporated into the primary multivariable model because of the limited sample size and the risk of model overfitting. Residual confounding related to these intrapartum factors therefore cannot be completely excluded. Additionally, as a single-center study, its generalizability is limited, and the sample size is insufficient to reliably evaluate rare but clinically significant adverse events (e.g., severe postpartum hemorrhage, major maternal complications, or serious neonatal adverse outcomes), reducing the ability to draw definitive conclusions regarding safety endpoints. Furthermore, individual variations in anesthetic dosing and maintenance strategies, oxytocin initiation dose and infusion rate, and labor management may have introduced treatment heterogeneity and affected result consistency. Although oxytocin was initiated after epidural analgesia had been established in the combination group, the detailed duration of oxytocin infusion and real-time dose adjustments were determined by clinical conditions and may not have been fully captured in a standardized manner in this retrospective analysis. The relatively short follow-up period also precludes comprehensive assessment of maternal and neonatal mid- to long-term outcomes. In addition, patient satisfaction, rescue analgesic requirements, and detailed evaluation of inadequate analgesia were not systematically available, which may limit interpretation of the pain-related outcomes. To enhance the robustness of conclusions, future studies should prioritize prospective, multicenter randomized controlled trials (RCTs) to validate the findings of this study. Adequate sample size calculation—particularly for safety endpoints—should be performed, and stratified or cluster randomization may be considered to balance key covariates. Whenever feasible, blinding or independent outcome adjudication should be applied to minimize bias. If RCTs cannot be conducted in the short term, improvements in observational study design—such as prospective cohort studies with systematic collection of potential confounders, propensity score matching/weighting, or instrumental variable analysis—combined with standardized multicenter epidural analgesia and oxytocin administration protocols, unified outcome definitions, and extended follow-up, can help reduce bias, enhance statistical power, and comprehensively evaluate both the clinical associations and safety profile of the intervention.

## 5 Conclusion

This retrospective study suggests that epidural analgesia combined with low-dose oxytocin was associated with lower labor pain scores, shorter labor duration, and a higher rate of vaginal delivery in primiparous women, without a detectable increase in common short-term maternal or neonatal adverse events. These findings support the potential clinical feasibility of the combined regimen in selected nulliparous women, particularly in relation to measured pain scores and delivery outcomes. However, because of the retrospective non-randomized design and clinical indication-based oxytocin administration, the observed associations should not be interpreted as evidence of causality or a confirmed synergistic effect between oxytocin and epidural analgesia. Further prospective randomized controlled trials are warranted to confirm its long-term efficacy and safety.

## Data Availability

The original contributions presented in the study are included in the article/Supplementary Material, further inquiries can be directed to the corresponding author/s.
